# Poor histological lesions in IgA nephropathy may be reflected in blood and urine peptide profiling

**DOI:** 10.1186/1471-2369-14-82

**Published:** 2013-04-11

**Authors:** Fredzzia Graterol, Maribel Navarro-Muñoz, Meritxell Ibernon, Dolores López, Maria-Isabel Troya, Vanessa Pérez, Josep Bonet, Ramón Romero

**Affiliations:** 1 Department of Nephrology, Germans Trias i Pujol Hospital, Universitat Autònoma de Barcelona, Esfera UAB, Carretera de Canyet, s/n 08916-Badalona, Barcelona, Spain; 2 Health Sciences Research Institute “Germans Trias i Pujol”, Universitat Autònoma de Barcelona, Esfera UAB, Carretera de Can Ruti, Camí de les Escoles, s/n, 08916-Badalona, Barcelona, Spain; 3 Departament of Pathology, Germans Trias i Pujol Hospital, Universitat Autònoma de Barcelona, Esfera UAB, Carretera de Canyet, s/n 08916-Badalona, Barcelona, Spain

## Abstract

**Background:**

IgA nephropathy (IgAN) is the most common primary glomerulonephritis worldwide, leading to renal failure in 15% to 40% of cases. IgAN is diagnosed by renal biopsy, an invasive method that is not risk-free. We used blood and urine peptide profiles as a noninvasive method of linking IgAN-associated changes with histological lesions by Oxford classification.

**Methods:**

We prospectively studied 19 patients with biopsy-proven IgAN and 14 healthy subjects from 2006 to 2009, excluding subjects with crescentic glomerulonephritis and collecting clinical and biochemical data at the time of diagnosis and during follow-up (24 months). Histological lesions were evaluated by Oxford classification. Proteomic analysis was performed by combining magnetic bead (MB) technology and mass spectrometry (MALDI-TOF MS) to obtain peptide profiles. Doubling of serum creatinine was considered a variable of poor renal prognosis.

**Results:**

We identified 55 peptides—13 in serum, 26 in plasma, and 16 in urine—that differentiated IgAN patients from healthy subjects. A significant association was noted between serum/plasma and urine peptides and histological findings—ie, tubulointerstitial damage, segmental glomerulosclerosis, and endocapillary injury.

We also identified 3 peptides—corresponding to bradykinin, uromodulin, and alpha-1-antitrypsin—that were associated with severity of lesions, such as tubulointerstitial damage and segmental glomerulosclerosis.

Moreover, blood peptides with *m/z* 2953, 5337, 9287, and 9289 and urine peptides with *m/z* 1769, 1898, 1913, 1945, 2491, 2756, 2977, 3004, 3389, and 4752 correlated significantly with poor renal function.

**Conclusions:**

In patients with IgAN, the use of noninvasive approaches, such as blood and urine proteomics, can provide valuable information beyond that of standard diagnostic techniques, allowing us to identify blood and urine peptide profiles that are associated with poor histological lesions in IgAN patients.

## Background

IgA nephropathy (IgAN) is the most common primary glomerulonephritis worldwide and is a significant cause of renal disease, leading to end-stage renal disease (ESRD) in 15% to 40% of patients after 20–25 years of follow-up [1]. For this reason, methods must be developed to make an early diagnosis. Several clinical risk factors, such as male gender, hypertension, increased serum creatinine level, proteinuria >1 g/day, and absence of hematuria, are associated with a poor prognosis [2]. Further, histopathological findings at the time of diagnosis, such as glomerulosclerosis and chronic tubulointerstitial damage, are also predictors of poor renal outcome [3-5].

A diagnosis of IgAN is biopsy-proven, based on pathological criteria that include the presence of diffuse mesangial IgA deposits by immunofluorescence. The Oxford classification is a recent histological classification system that is based on 4 scores: interstitial fibrosis/tubular atrophy (IFTA), segmental glomerulosclerosis, endocapillary hypercellularity, and mesangial hypercellularity [6,7]. This new assessment has demonstrated that each variable correlates with clinical outcome [8,9].

In the past decade, proteomics has been applied extensively to various fields of medicine, including nephrology [10-15]—particularly in urine, because it can be obtained noninvasively, allowing one to identify glomerular kidney disease (GKD)-related markers [16-26]. Profiling methods are gaining popularity in the quest for new putative biomarkers for glomerular disorders [27-35]. Magnetic bead (MB)-based fractionation methods, coupled with MALDI-TOF MS, were introduced recently as a urinary peptide profiling strategy [36-38] and have emerged as a suitable platform for rapid, high-throughput analysis.

The main objective of our study was to identify peptide profiles in blood and urine that are associated with IgAN and its histological lesions.

## Methods

### Study population

This prospective study was performed between June 2006 and November 2009 in the Nephrology Department, Germans Trias i Pujol Hospital (Barcelona, Spain). All procedures were conducted per the Declaration of Helsinki of 1971, as revised in 2008. The Clinical Research Ethics Committee of Germans Trias i Pujol Hospital approved the study protocol, and all patients gave written informed consent to participate.

### Study procedures

For inclusion, patients had to be aged older than 18 years; show clinical signs of renal disease, such as proteinuria with stable renal function or various degrees of renal failure and the presence of hematuria; and be indicated for renal biopsy. Only patients with primary IgAN were included. Patients with crescentic glomerulonephritis were excluded.

Healthy subjects were used as controls to establish a normal peptide profile.

### Measurement of clinical and biochemical parameters

The following clinical data were collected at the time of diagnosis: age, gender, history of hypertension, diabetes mellitus, and dyslipidemia. Biochemical data were collected at the time of study and after 2 years of follow-up: serum levels of creatinine, uric acid, albumin, and cholesterol; and proteinuria. Statin therapy and antihypertensive treatments that were based on renin-angiotensin-aldosterone system (RAAS)-blocking drugs, such as angiotensin-converting enzyme inhibitors (ACEI) and angiotensin II receptor blockers (ARB), were recorded (Table 1).

**Table 1:** Demographic, clinical, and biochemical data of IgAN patients

	**IgAN patients**	
	**At the time of initial biopsy**	**At the end of follow-up**
Number of patients	19	
Age, years	42 (33–52)	44 (35–54)
Gender (Male), n (%)	15 (79)	
Hypertension (Yes), n (%)	12 (63)	
Diabetes (Yes), n (%)	1 (5)	
Dyslipidemia (Yes), n (%)	9 (47)	
Serum creatinine, mg/dl	2.00 (1.40-2.57)	3.16^a^ (1.26-6.18)
Uric acid, mg/dl	7.40 (5.45-8.30)	7.40 (6.55-9.20)
Serum albumin, g/l	34.00 (31.00–39.00)	40.00^a^ (36.75–42.40)
Cholesterol, mg/dl	194.00 (157.00–221.75)	147.00^a^ (131.50-213.00)
Proteinuria, g/24 hours	2.28 (1.20-4.26)	1.30 (0.52-3.27)
RAAS blockade (Yes), n (%)	7 (37)	17 (89)
ACEI	5 (26)	11(58)
ARB	1 (5)	3(16)
ACEI plus ARB	1 (5)	3(16)
Statins (Yes), n (%)	4 (21)	9 (47)

Data are expressed as median and ranges. ^a^Significant Wilcoxon test (*P* < 0.05) in IgAN patients between the time of initial biopsy and the end of follow-up. RAAS: renin-angiotensin-aldosterone system; ACEI: angiotensin-converting enzyme inhibitors; ARB: angiotensin II receptor blockers.

### Renal biopsy

Percutaneous renal biopsies were performed and processed routinely for light, immunofluorescence, and electron microscopy per standard procedures.

Light microscopy sections were stained with hematoxylin/eosin, Schiff’s periodic acid, methenamine silver, Masson trichrome, and Congo red.

At the time of the biopsy, a single pathologist reviewed all renal biopsy slides and scored the pathological variables per the Oxford classification as follows: IFTA ≤25% (T0), 26% to 50% (T1) or >50% (T2); segmental glomerulosclerosis absent (S0) or present (S1); endocapillary hypercellularity absent (E0) or present (E1); and mesangial hypercellularity ≤0.5 (M0) or > 0.5 (M1) [6].

### Blood samples

Blood samples were collected from the participants on the same day that the renal biopsy was performed. Serum and plasma-EDTA samples were obtained by centrifugation at 2200 g for 10 min, aliquoted to avoid freeze-thaw cycles, and stored at −80°C until use.

### Urine samples

Fresh, first-morning urine samples were collected from the participants on the same day that the renal biopsy was performed. Briefly, urine samples were centrifuged at 2100 g for 30 min at 4°C to remove particulate material and cellular debris. The supernatant was adjusted to 6.5 pH with NH_4_HCO_3_ (1 M) to minimize precipitation during storage, aliquoted to avoid freeze-thaw cycles, and stored at −80°C until use [36]. Protease inhibitors were not added. Normalization of the samples to urinary protein concentration was not necessary to analyze peptide profiles [38].

### Peptide enrichment and identification by MALDI-TOF MS analysis

Peptides in serum and plasma (80 μl) and urine supernatant (110 μl) were extracted using MB. The profiling kit that we used to analyze blood samples was the MB-IMAC Cu kit (Bruker Daltonics, Bremen, Germany), which is based on the affinity of peptides to immobilized copper ions on the surface of MB. In the urine samples, the beads that we used were coated with C18 aliphatic chains, wherein peptides were captured by hydrophobic interactions (Dynabeads® RP-C18, Invitrogen, the Netherlands).

Each serum, plasma, and urine sample was processed in duplicate, and each duplicate was spotted twice on the MALDI target (AnchorChip 600/384, Bruker Daltonics). Thus, 4 MALDI spectra were obtained for each sample, acquired for *m/z* values from 1000 to 10,000. ClinProTools (v2.0; Bruker Daltonics) was used to analyze peptide profiles, subtract baseline values, and normalize the spectra. Further details on the MALDI-TOF MS analysis are described in our previous report [38,39].

### Statistical analysis

Normality of the variables was assessed by Kolmogorov-Smirnov test*.* Continuous variables were expressed as median and range and were compared by Mann–Whitney *U*-test or Kruskal–Wallis test, as appropriate. Wilcoxon test was performed for paired samples as required to assess differences at the end of follow-up. Categorical variables were analyzed using chi-square or Fisher’s exact probability test. Associations between variables were assessed using the Spearman correlation coefficient.

Statistical analyses were performed using SPSS, v15.0 (SPSS Inc., Chicago, IL). A two-tailed *p*-value of <0.05 was considered to be statistically significant.

## Results

### Patients

Nineteen IgAN patients were enrolled, and 14 healthy subjects were included in the study as controls. The baseline clinical and biochemical characteristics of the IgAN patients are summarized in Table 1. Healthy subjects ranged in age from 26 to 41 years (median 30 years); 6 were male (43%). The entire control group had normal renal function without hematuria or proteinuria, and none was treated.

Significant differences were observed between IgAN patients and healthy subjects with regard to age and renal function but not gender. Also, we compared clinical and biochemical variables at the time of biopsy with those at the end of the follow-up (24 months) (Table 1). During the follow-up, 5 patients (26.3%) with impaired serum creatinine doubled their serum creatinine levels. In contrast, proteinuria levels fell, albeit insignificantly. Further, serum cholesterol and serum albumin levels differed significantly between baseline and at the end of the follow-up.

### Identification of IgAN-related peptides in blood and urine

The blood and urinary peptide profiles of IgAN patients differed significantly from those of healthy subjects; using Clinprotools, we obtained 13 differential peaks in serum, 26 in plasma, and 16 in urine that discriminated IgAN patients from healthy subjects and achieved statistical significance (Additional file 1). Two peaks in plasma and 6 peaks in urine were identified in our earlier reports [38,39].

Our results suggest that peptides that were detected in plasma by MALDI-TOF at *m/z =* 1063 and *m/z =* 1898 correspond to bradykinin (KNG1) and complement factor C4 (C4A), respectively [39]. Similarly, peptides that were detected in urine by MALDI-TOF at *m/z =* 1898 and 1913 corresponded to uromodulin peptides (UMOD); those at *m/z =* 1945, 2392, and 2505 corresponded to alpha-1-antitrypsin peptides (A1AT); and that at *m/z =* 2714 corresponded to beta-2-microglobulin (B2M) [38].

### Association between IgAN-related peptides and histological lesions by Oxford classification

The histological lesions of the IgAN patients are summarized in Table 2.

**Table 2:** Scores of histological lesions by Oxford classification in IgAN patients

**Histological lesions**	**IgAN patients**
IFTA, n (%)	
T0	6 (32)
T1	8 (42)
T2	5 (26)
Segmental glomerulosclerosis, n (%)	
S0	3 (16)
S1	16 (84)
Endocapillary hypercellularity, n (%)	
E0	12 (63)
E1	7 (37)
Mesangial hypercellularity, n (%)	
M0	0 (0)
M1	19 (100)

Interstitial fibrosis/tubular atrophy (IFTA).

The blood and urine peptides of IgAN patients were associated with IFTA lesions (Additional file 2). In evaluating tubulointerstitial damage T2 (>50%) in patients with IgAN, we noted that serum peptides at *m/z* 5966 and 9289 increased in peak area compared with the other 2 groups (T0, T1). Moreover, serum peptides at *m/z* 1466, 1617, 3193, 3264, 5337, 5889, and 5905 increased in peak area with respect to T1.

Similarly, plasma peptides at *m/z* 2661 and 2790 rose in peak area versus the other 2 groups (T0, T1), but plasma peptides at *m/z* 1063 and 1078 decreased in peak area in IgAN patients with tubulointerstitial damage T2 compared with subjects with T0 and T1.

**Figure 1:**
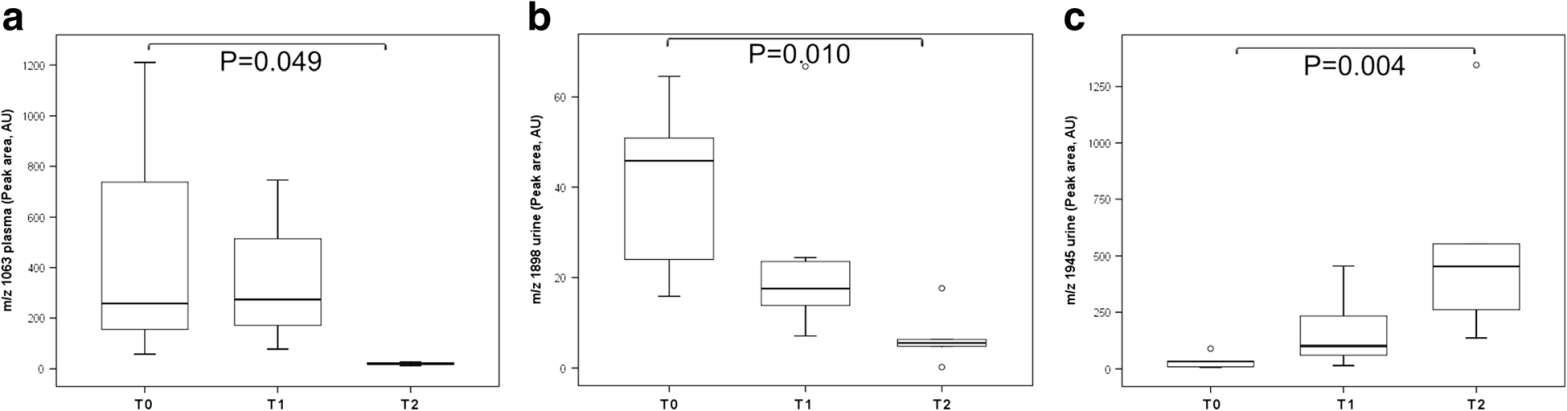
**Differential expression of bradykinin, uromodulin, and alpha-1-antytripsin peptides by interstitial fibrosis/tubular atrophy (IFTA) lesion.** Box plot of differential plasma expression of *m/z* 1063 (**a**) and urine expression of *m/z* 1898 (**b**) and *m/z* 1945 (**c**) in IgAN patients without (T0) or with (T1, T2) interstitial fibrosis/tubular atrophy (IFTA) lesions. Outliers are open circles. AU = Arbitrary units.

In urine, peptides at *m/z* 1945 and 2491 increased in peak area in IgAN patients with T2 tubulointerstitial damage (>50%), but peptides at *m/z* 1769, 1898, 2977, 3004, 3389, 3406, 4658, and 4752 fell compared with the T0 and T1 groups. Further, as shown in Figure 1a-c, bradykinin peptide (*m/z* 1063) and UMOD peptide (*m/z* 1898) decreased in parallel with the progression of tubulointerstitial damage. But, A1AT peptide increased proportionally to tubulointerstitial damage.

**Figure 2:**
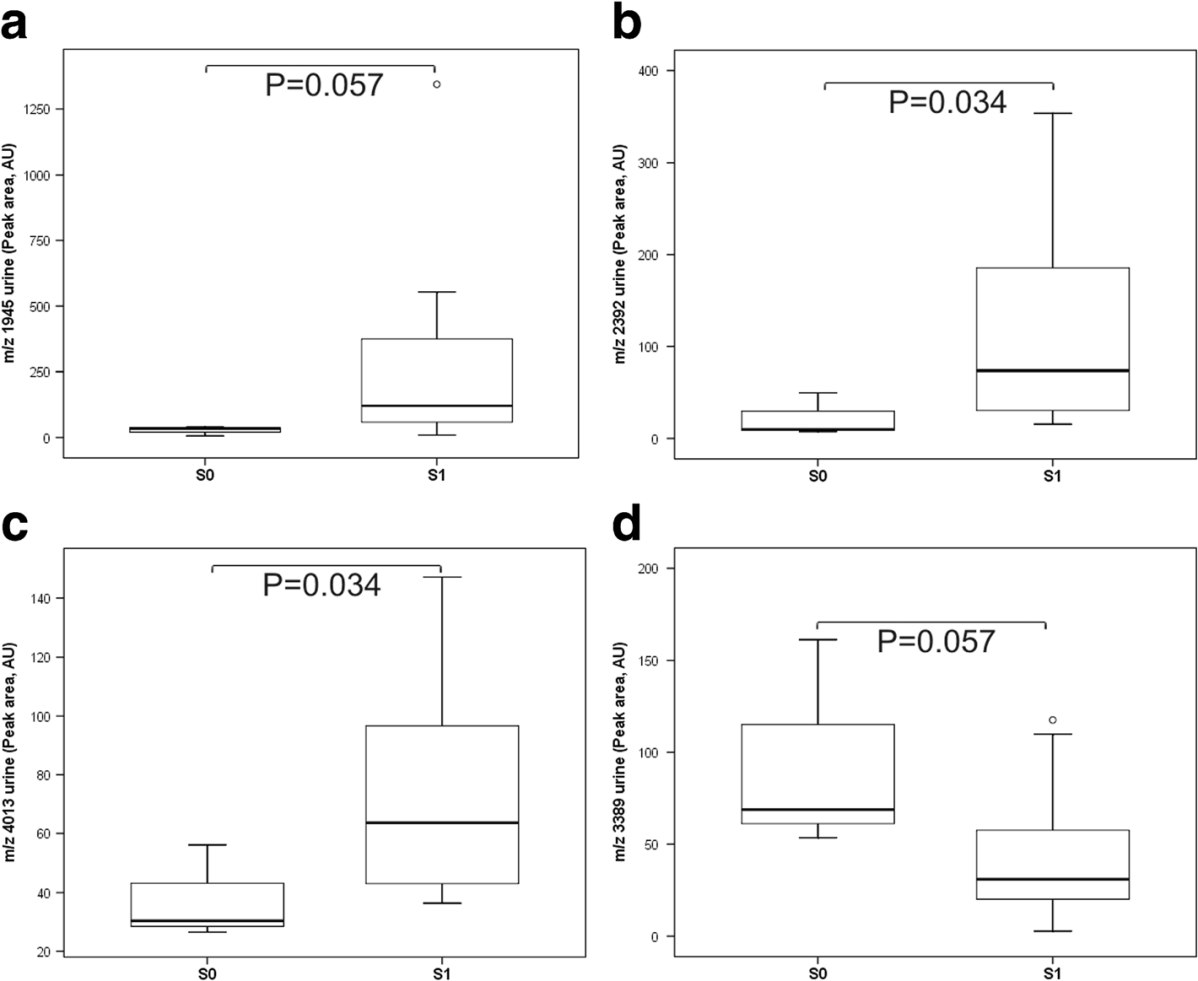
**Differentially expressed peptide peaks in urine by segmental glomerulosclerosis lesion.** Box plot of differential urine expression of *m/z* 1945 (**a**), *m/z* 2392 (**b**), *m/z* 4013 (**c**), and *m/z* 3389 (**d**) in IgAN patients without (S0) or with (S1) segmental glomerulosclerosis lesions. Outliers are open circles. AU = Arbitrary units.

As shown in Figure 2a-d, urine peptides at *m/z* 1945, 2392, and 4013 increased in peak area in IgAN patients with S1 lesions, but the peptide at *m/z* 3389 fell in peak area compared with IgAN patients with S0 lesions. No associations were observed between segmental glomerulosclerosis and blood peptides.

**Figure 3:**
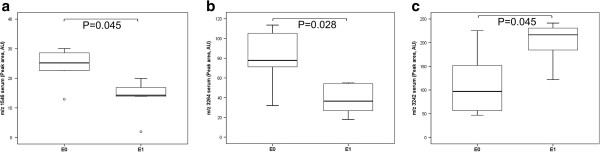
**Differentially expressed peptide peaks in serum by endocapillary hypercellularity lesion.** Box plot of differential serum expression of *m/z* 1546 (**a**), 3264 (**b**), and 3242 (**c**) in IgAN patients without (E0) or with (E1) endocapillary hypercellularity lesions. Outliers are open circles. AU = Arbitrary units.

**Figure 4:**
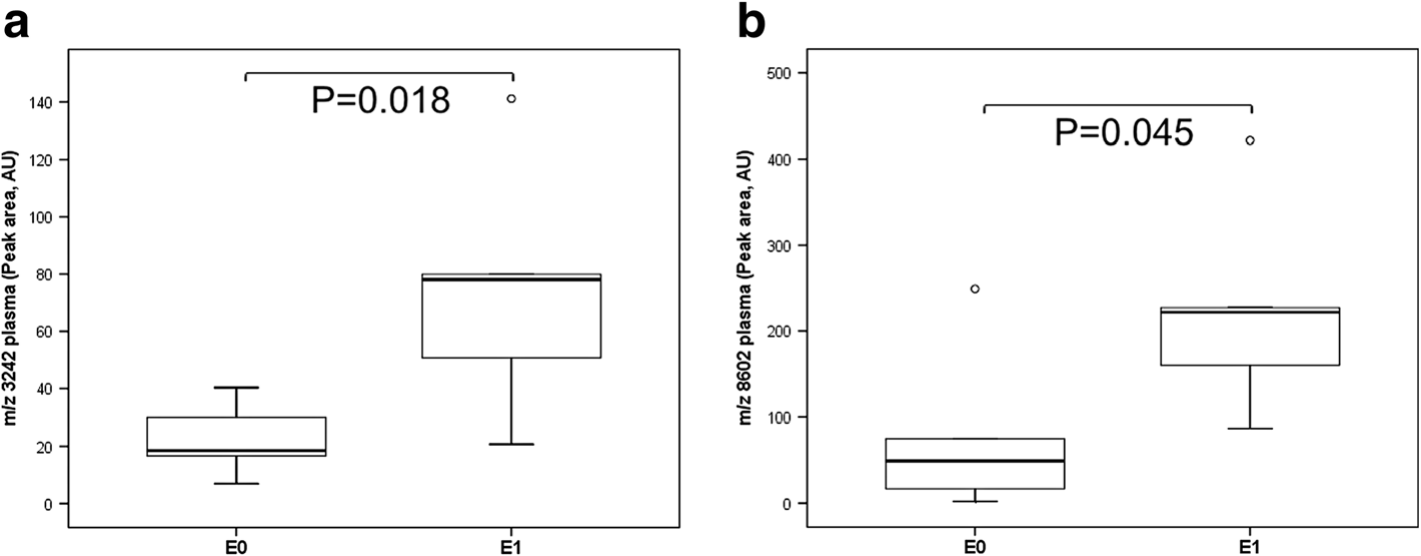
**Differentially expressed peptide peaks in plasma by endocapillary hypercellularity lesion.** Box plot of differential plasma expression of *m/z* 3242 (**a**) and *m/z* 8602 (**b**) in IgAN patients without (E0) or with (E1) endocapillary hypercellularity lesions. Outliers are open circles.

A lack of endocapillary hypercellularity (E0) was associated with increased peak areas of peptides at *m/z* 1546 and 3264, but the *m/z* 3242 peptide declined in peak area in the serum of IgAN patients (Figure 3a-c). Moreover, E0 correlated with decreased peak area of peptides at *m/z* 3242 and *m/z* 8602 in the plasma of IgAN patients compared with endocapillary hypercellularity (E1) (Figure 4a-b). No associations were observed in the urine peptides.

No associations were observed between blood or urine peptides and mesangial hypercellularity in IgAN patients.

### Association between peptides and doubling of serum creatinine in IgAN

Serum peptides at *m/z* 2953, 5337, and 9289 and plasma peptides at *m/z* 9287 were linked with the doubling of serum creatinine in IgAN patients. Also, urine peptides at *m/z* 1769, 1898, 1913, 1945, 2491, 2756, 2977, 3004, 3389, and 4752 correlated with poor creatinine levels in IgAN patients (Table 3).

**Table 3:** Relationship between doubling of serum creatinine and peptide peaks

**Biofluid**	**Peptides ( *m/z *)**	**R**	p*
Serum	2953	0.624	0.040
	5337	−0.615	0.044
	9289	0.711	0.014
Plasma	9287	0.615	0.044
Urine	1769	−0.718	0.001
	1898	−0.734	< 0.001
	1913	−0.598	0.007
	1945	0.603	0.006
	2491	0.465	0.045
	2756	−0.443	0.058
	2977	−0.564	0.012
	3004	−0.631	0.004
	3389	−0.529	0.020
	4752	−0.530	0.020

### Association between peptides and clinical parameters

Urinary UMOD-derived peptides (*m/z* 1898, 1913) correlated inversely between baseline serum creatinine, and *m/z* 1913 also correlated inversely with proteinuria. Urinary A1AT-derived peptides (*m/z* 1945, 2392 and 2505) showed a positive association with proteinuria. Also, *m/z* 1945 showed a positive correlation with serum creatinine.

Most patients had varying degrees of hematuria at diagnosis; thus, it was not possible to compare the influence of hematuria on peptide profile changes.

## Discussion

In this preliminary prospective study, peptide patterns in blood and urine were observed in patients with IgAN that allowed us to differentiate them from healthy subjects. Bradykinin peptide and a fragment of complement C4a were identified in blood samples, as were UMOD, A1AT, and B2-microglobulin peptides in urine. Moreover, these peptides were associated with poor histological lesions in IgAN patients, particularly with tubulointerstitial damage and segmental glomerulosclerosis.

In IgAN, much effort has been aimed to correlate a wide range of histological lesions with clinical outcome. In this report, we used the Oxford classification to explore IgAN-associated changes from blood and urine peptide profiles, because it is the most recent pathological classification system and was developed as a reproducible method of predicting the risk of disease progression with regard to clinical outcome and histological lesions.

Recently, new methods have been described to identify biological biomarkers in various fluids using proteomic techniques. In our previous report [38], we demonstrated that MB-based profiling and MALDI-TOF MS identify disparities in urine between GKD patients and controls, suggesting that establishment of a differential peptide profile is the initial step toward classifying GKD. In the current study, the majority of peptides, specifically high levels of an A1AT-derived peptide (*m/z* 1945) and low levels of a UMOD-derived peptide (*m/z* 1898) and bradykinin (*m/z* 1063), was linked to tubulointerstitial damage.

Alpha-1-antitrypsin protects the extracellular matrix from neutrophil attack through its anti-inflammatory and anti-apoptotic effects. Kwak et al. [40] reported increased levels of A1AT peptides in kidney tissue and urine in IgAN patients compared with healthy subjects. The authors speculated that renal tubular epithelial cells produce A1AT in response to tubulointerstitial damage. Consistently, our results reinforce the suggestion that high levels of A1AT in urine might constitute a response to the inflammatory process in GKD. Likewise A1AT has a function in tubulointerstitial injury; we noted an inverse association between UMOD-derived peptides and tubulointerstitial lesions, consistent with other groups that have described low urinary UMOD levels in association with tubular atrophy and interstitial infiltration in renal biopsies [41].

Uromodulin is a glycoprotein that is expressed on renal tubular epithelial cells and is the most abundant protein in urine. Recent studies have implicated UMOD in chronic kidney disease [41-43]. In a previous report, we observed an inverse correlation between UMOD peptides, serum creatinine, and proteinuria in patients with GKD [38].

Further, in IgAN, Wu et al. [37] described a urine peptide profile, noting downregulation of peptide that corresponded to UMOD, allowing them to discriminate IgAN from healthy subjects. In the current study, our peptide profile could be a biomarker of histological lesions but is not specific for IgAN.

Rocchetti et al. [23] reported a significant decrease in the urinary excretion of kininogen in IgAN patients, particularly in unresponsive ACEI therapy patients. The authors speculated that this difference reflects the severity of renal damage in IgAN patients. Similarly, we found a decreased in kininogen-derived peptide bradykinin (*m/z* 1063) in plasma from IgAN patients with severe tubulointerstitial damage T2. But, we could not demonstrate this effect because most patients did not receive ACEI or ARB agents at baseline. Thus, we could not compare the effects of these drugs on peptide profiles.

Bradykinin is a nonapeptide that is derived from the kininogen protein, a robust agonist of the bradykinin 2 receptor, enhancing the production of nitric oxide and prostaglandins. Potent renal vasodilator, antithrombotic and antifibrotic effects of kinins have been observed recently in diabetic nephropathy [44]. Furthermore, upregulation of bradykinin receptor has been described to mediate the progression of focal segmental glomerulosclerosis [45].

In a previous report, Kang et al. [9] observed that segmental glomerulosclerosis and IFTA reflect chronic damage, which can be used to predict the long-term prognosis of patients with IgAN. Our findings suggest that a rise in peptides at *m/z* 1945, 2392, and 4013 and a decline in the peptide at *m/z* 3389 observed in patients with S1 reflect chronic glomerular injury.

Endocapillary and mesangial hypercellularity lesions were not associated with renal outcome in the original Oxford cohorts, although few reports have reported such findings. In our study, we identified 5 peptides—in serum at *m/z* 1546, 3264, and 3242 and in plasma at *m/z* 3242, 8602—of which the plasma and serum peptides at *m/z* 3242 and plasma peptide at *m/z* 8602 increased and serum peptides at *m/z* 1546 and 3264 decreased in the presence of endocapillary hypercellularity.

Further, many IgAN patients showed mesangial hypercellularity—this score is defined as the number of mesangial cells per mesangial area. Previous studies have shown that mesangial hypercellularity correlates with an active lesion; perhaps patients with M1 have a good response to treatment.

A limitation of this study is that we could not confirm that peptides of bradykinin, UMOD and A1AT are related specifically to IgAN, because our population was in various stages of kidney disease and degrees of interstitial injury. Interestingly, we found a relationship between peptide profiles in the serum, plasma, and urine of IgAN patients with the doubling of serum creatinine levels—particularly, in the urine samples with UMOD and A1AT peptides. Based on these findings, we propose that this peptide profile is a potential biomarker predictor of renal outcome, but we can not exclude the possibility that these peptides constitute a biomarker of chronic kidney diseases. It is necessary to expand this population study to other types of GKD to mitigate the limitations on its reproducibility and thus obtain a specific peptide profile.

Similarly, our patients had mesangial hypercellularity, and all were classified as M1. This feature of our study population did not allow us to evaluate the influence of this injury on peptide profiles. Finally, identifying peptides in biological fluids is critical in determining their function in the disease process.

## Conclusions

IgAN is a glomerular disease, the pathogenic mechanism of which remains unknown. Proteomic analysis allows us to obtain a peptide profile that can enhance the diagnosis and prognosis of this glomerular disease. Further studies with more patients are needed to determine the function of bradykinin, UMOD, and A1AT peptides in the pathogenesis of IgAN.

## Competing interests

The authors declare no potential conflicts of interest with respect to the authorship or publication of this article. The results presented in this paper have not been published previously in whole or in part, except in abstract format.

## Authors’ contributions

FG: participated in the conception and design of the study, carried out the collection of samples, clinical data management, statistical analysis and involved in drafting the manuscript and revising it critically for important intellectual content. MNM: participated in the conception and design of the study, carried out the collection of samples, analysis and interpretation of proteomic data, statistical analysis and involved in drafting the manuscript and revising it critically for important intellectual content. MI: participated in the conception and design of the study, carried out the collection of samples, clinical data management, statistical analysis and involved in drafting the manuscript and revising it critically for important intellectual content. DL: carried out the histological analysis. MIT: helped to perform the statistical analysis. VP: participated in the collection of samples. JB: involved in revising the manuscript critically for important intellectual content and has given final approval of the version to be published. RR: made substantial contributions to conception and design, involved in revising the manuscript critically for important intellectual content and has given final approval of the version to be published. All authors read and approved the final manuscript.

## Pre-publication history

The pre-publication history for this paper can be accessed here:

http://www.biomedcentral.com/1471-2369/14/82/prepub

## Supplementary Material

Additional file 1 Differentially expressed peptide peaks between IgAN patients and healthy subjects. Click here for file

Additional file 2 Differentially expressed peptide peaks by interstitial fibrosis/tubular atrophy (IFTA) lesion. Click here for file
