# Supplementary material for: Poor histological lesions in IgA nephropathy may be reflected in blood and urine peptide profiling
Source: BMC Nephrol. 2013 Apr 11;14:82. doi: 10.1186/1471-2369-14-82 (PMC3637490; doi:10.1186/1471-2369-14-82)
Supplement: Additional file 1 — Differentially expressed peptide peaks between IgAN patients and healthy subjects. [file 1471-2369-14-82-S1.doc]

**Additional file 1 – Differentially expressed peptide peaks between IgAN patients and healthy subjects**

| **Biofluid** | **Peptide MH^+^ measured by MALDI-TOF** | **Swiss-Prot accession** | **Protein abbreviation** | **Fold-change^*^** | ***P* value*^**^*** |
| --- | --- | --- | --- | --- | --- |
| Serum | 1063 | n.a. | n.a. | +3.23 | *0.008* |
|  | 1079 | n.a. | n.a. | +2.77 | *0.046* |
|  | 1532 | n.a. | n.a. | -0.63 | *0.040* |
|  | 1618 | n.a. | n.a. | -0.53 | *0.052* |
|  | 1898 | n.a. | n.a. | -0.62 | *0.002* |
|  | 2023 | n.a. | n.a. | +2.03 | *0.010* |
|  | 2864 | n.a. | n.a. | -0.45 | *0.014* |
|  | 2934 | n.a. | n.a. | -0.57 | *0.003* |
|  | 3159 | n.a. | n.a. | -0.50 | *0.003* |
|  | 3193 | n.a. | n.a. | -0.54 | *0.007* |
|  | 4283 | n.a. | n.a. | -0.60 | *0.026* |
|  | 5337 | n.a. | n.a. | -0.80 | *0.010* |
|  | 5967 | n.a. | n.a. | -0.51 | *0.008* |
| Plasma | 1049 | n.a. | n.a. | -0.24 | *0.001* |
|  | 1063^a^ | P01042 | KNG1 | -0.25 | *0.003* |
|  | 1079 | n.a. | n.a. | -0.16 | *0.010* |
|  | 1084 | n.a. | n.a. | -0.08 | *<0.001* |
|  | 1100 | n.a. | n.a. | -0.15 | *0.001* |
|  | 1360 | n.a. | n.a. | -0.35 | *0.002* |
|  | 1532 | n.a. | n.a. | -0.33 | *0.001* |
|  | 1607 | n.a. | n.a. | +4.62 | *0.001* |
|  | 1779 | n.a. | n.a. | +2.48 | *<0.001* |
|  | 1848 | n.a. | n.a. | +1.55 | *0.003* |
|  | 1866 | n.a. | n.a. | +4.05 | *0.001* |
|  | 1883 | n.a. | n.a. | +3.03 | *0.001* |
|  | 1898^a^ | P0C0L4 | C4A | +2.52 | *0.001* |
|  | 1936 | n.a. | n.a. | +6.71 | *<0.001* |
|  | 1960 | n.a. | n.a. | +1.75 | *0.014* |
|  | 2008 | n.a. | n.a. | +6.38 | *<0.001* |
|  | 2023 | n.a. | n.a. | +8.22 | *<0.001* |
|  | 2312 | n.a. | n.a. | -0.11 | *0.001* |
|  | 2358 | n.a. | n.a. | -0.20 | *0.001* |
|  | 2375 | n.a. | n.a. | -0.31 | *0.001* |
|  | 2486 | n.a. | n.a. | -0.24 | *0.007* |
|  | 2863 | n.a. | n.a. | -0.14 | *<0.001* |
|  | 3242 | n.a. | n.a. | -0.39 | *0.033* |
|  | 4576 | n.a. | n.a. | -0.21 | *0.009* |
|  | 4965 | n.a. | n.a. | +20.34 | *0.002* |
|  | 4981 | n.a. | n.a. | +22.73 | *0.002* |
| Urine | 1898^b^ | P07911 | UMOD | -0.21 | *<0.001* |
|  | 1913^b^ | P07911 | UMOD | -0.09 | *<0.001* |
|  | 1945^b^ | P01009 | A1AT | +9.66 | *<0.001* |
|  | 2378 | n.a. | n.a. | +2.14 | *0.006* |
|  | 2392^b^ | P01009 | A1AT | +3.62 | *0.001* |
|  | 2491 | n.a. | n.a. | +3.10 | *<0.001* |
|  | 2505^b^ | P01009 | A1AT | +10.57 | *<0.001* |
|  | 2714^b^ | P61769 | B2M | -0.28 | *0.001* |
|  | 2977 | n.a. | n.a. | -0.36 | *<0.001* |
|  | 3004 | n.a. | n.a. | -0.31 | *0.001* |
|  | 3389 | n.a. | n.a. | -0.31 | *<0.001* |
|  | 3406 | n.a. | n.a. | -0.27 | *<0.001* |
|  | 4013 | n.a. | n.a. | +1.26 | *0.049* |
|  | 4294 | n.a. | n.a. | -0.59 | *0.049* |
|  | 4658 | n.a. | n.a. | -0.43 | *<0.001* |
|  | 4752 | n.a. | n.a. | -0.30 | *<0.001* |

^a^Bradykinin (KNG1) and complement factor C4 (C4A) peptides identified by MALDI-TOF/TOF MS analysis, in ascending order by MH+ weight (Dalton), as reported^39^. ^b^Uromodulin (UMOD), alpha-1-antitrypsin (A1AT), and beta-2-microglobulin (B2M) peptides identified by HPLC-MS/MS analysis, in ascending order by MH+ weight (Dalton), as reported^38^. *Ratio between the median peak area of IgAN patients compared with healthy subjects. The (+) and (-) values are increased and decreased peak area values, respectively. **Significant Mann-Whitney U-test (P<0.05). UMOD: uromodulin; A1AT: alpha-1-antitrypsin; B2M: beta-2-microglobulin; KNG1: bradykinin; C4A: complement factor C4; n.a.: not available.
