# Supplementary material for: Poor histological lesions in IgA nephropathy may be reflected in blood and urine peptide profiling
Source: BMC Nephrol. 2013 Apr 11;14:82. doi: 10.1186/1471-2369-14-82 (PMC3637490; doi:10.1186/1471-2369-14-82)
Supplement: Additional file 2 — Differentially expressed peptide peaks by interstitial fibrosis/tubular atrophy (IFTA) lesion. [file 1471-2369-14-82-S2.doc]

**Additional file 2 – Differentially expressed peptide peaks by interstitial fibrosis/ tubular atrophy (IFTA) lesion**

| **Biofluid** | **Peptides (*m/z,* AU)** | **TO** | **T1** | **T2** | **p*** |
| --- | --- | --- | --- | --- | --- |
| Serum | 1466 | 14.59  (11.14-77.66) | 6.80  (2.17-9.65) | 61.44  (22.84-99.24) | 0.020 |
|  | 1617 | 28.47  (22.80-60.53) | 13.46  (8.93-15.51) | 44.50  (22.75-62.99) | 0.029 |
|  | 3193 | 58.13  (41.11-70.71) | 23.42  (14.69-32.80) | 38.35  (37.84-81.76) | 0.030 |
|  | 3264 | 76.38  (58.46-99.35) | 29.37  (20.14-35.33) | 74.01  (55.00-113.65) | 0.029 |
|  | 5337 | 220.49  (214.37-235.50) | 109.47  (54.45-164.65) | 191.20  (119.59-212.96) | 0.016 |
|  | 5889 | 248.44  (221.10-293.41) | 198.39  (52.45-246.57) | 283.06  (270.31-453.76) | 0.052 |
|  | 5905 | 1090.49  (918.54-1283.53) | 1042.24  (249.00-1205.96) | 1731.53  (1358.88-1891.18) | 0.041 |
|  | 5966 | 95.33  (86.83-140.95) | 117.38  (32.35-133.61) | 232.90  (177.94-343.53) | 0.049 |
|  | 9289 | 204.63  (87.63-321.87) | 371.75  (295.27-378.63) | 417.34  (349.94-485.85) | 0.036 |
| Plasma | 1063 | 257.50  (106.62-973.82) | 273.48  (124.69-630.09) | 20.02  (12.91-28.53) | 0.049 |
|  | 1078 | 135.27  (20.43-537.92) | 142.85  (62.26-283.79) | 8.96  (7.28-12.96) | 0.049 |
|  | 2661 | 25.25  (14.13-37.51) | 17.48  (6.85-25.13) | 60.39  (58.48-63.10) | 0.041 |
|  | 2790 | 22.05  (8.20-50.53) | 23.32  (4.29-66.61) | 119.78  (88.07-243.65) | 0.049 |
| Urine | 1769 | 101.49  (34.53-135.78) | 21.67  (7.15-66.39) | 7.79  (2.09-11.12) | 0.009 |
|  | 1898 | 45.84  (21.98-54.32) | 17.58  (12.82-24.01) | 5.48  (2.52-11.94) | 0.010 |
|  | 1945 | 33.04  (8.93-49.00) | 101.14  (50.88-263.48) | 453.78  (198.45-948.64) | 0.004 |
|  | 2491 | 24.68  (10.31-38.16) | 41.36  (22.17-92.58) | 172.68  (75.44-247.01) | 0.041 |
|  | 2977 | 50.50  (31.17-70.23) | 20.98  (13.71-36.43) | 6.31  (2.89-20.34) | 0.007 |
|  | 3004 | 51.62  (43.85-155.62) | 33.56  (24.25-68.36) | 11.74  (4.77-18.83) | 0.004 |
|  | 3389 | 61.07  (36.98-111.77) | 37.20  (27.25-59.45) | 18.82  (5.88-44.37) | 0.053 |
|  | 3406 | 30.88  (24.52-71.15) | 21.62  (12.57-44.37) | 6.38  (4.14-18.76) | 0.042 |
|  | 4658 | 30.38  (15.91-46.81) | 18.09  (6.56-27.95) | 5.58  (1.16-19.84) | 0.054 |
|  | 4752 | 35.51  (27.93-48.57) | 16.98  (8.29-28.06) | 16.11  (5.04-22.06) | 0.021 |

^*^Significant Kruskal–Wallis test (*P* < 0.05). The peak area values of different peptides have been represented in arbitrary units (AU).
